# Sox2 Level Is a Determinant of Cellular Reprogramming Potential

**DOI:** 10.1371/journal.pone.0067594

**Published:** 2013-06-18

**Authors:** Dong Wook Han, Natalia Tapia, Marcos J. Araúzo-Bravo, Kyung Tae Lim, Kee Pyo Kim, Kinarm Ko, Hoon Taek Lee, Hans R. Schöler

**Affiliations:** 1 Department of Stem Cell Biology, School of Medicine, Konkuk University, Seoul, Republic of Korea; 2 Institute of Functional Genomics, Konkuk University, Seoul, Republic of Korea; 3 Department of Cell and Developmental Biology, Max Planck Institute for Molecular Biomedicine, Münster, Germany; 4 University of Münster, Medical Faculty, Münster, Germany; Centro Cardiologico Monzino, Italy

## Abstract

Epiblast stem cells (EpiSCs) and embryonic stem cells (ESCs) differ in their *in vivo* differentiation potential. While ESCs form teratomas and efficiently contribute to the development of chimeras, EpiSCs form teratomas but very rarely chimeras. In contrast to their differentiation potential, the reprogramming potential of EpiSCs has not yet been investigated. Here we demonstrate that the epiblast-derived pluripotent stem cells EpiSCs and P19 embryonal carcinoma cells (ECCs) exhibit a lower reprogramming potential than ESCs and F9 ECCs. In addition, we show that the low reprogramming ability is due to the lower levels of *Sox2* in epiblast-derived stem cells. Consistent with this observation, overexpression of *Sox2* enhances reprogramming efficiency. In summary, these findings suggest that a low reprogramming potential is a general feature of epiblast-derived stem cells and that the *Sox2* level is a determinant of the cellular reprogramming potential.

## Introduction

Pluripotency has been recently classified into two distinct states namely, naïve and primed pluripotency [1–3]. The ground naïve pluripotent state refers to cells, such as embryonic stem cells (ESCs), that can form teratomas and contribute to chimeras. In contrast, primed pluripotency occurs in cells, such as epiblast stem cells (EpiSCs), that can form teratomas but can rarely form chimeras [1–3]. Furthermore, leukemia inhibitory factor (LIF) but basic fibroblast growth factor (bFGF) and Activin are required to maintain self-renewal in the naïve and primed pluripotent state, respectively. In female cells, both X chromosomes remain activated in the naïve ESCs while one chromosome is randomly inactivated in the primed EpiSCs [1–3]. However, in spite of these differences, the transcription factors *Oct4*, *Sox2*, and *Nanog* are crucial components of the regulatory circuit in both pluripotency states [4–6]. Moreover, recent reports have confirmed that *Oct4* and *Sox2* together with *Klf4* and *c-Myc* can induce naïve or primed pluripotency in somatic cells depending on the applied culture conditions [7–9].

P19 is an embryonic carcinoma cell (ECC) line derived from a 7.5 days post coitum (d.p.c.) embryo that was transplanted into the testis [10]. P19 ECCs maintain a male euploid karyotype and can differentiate into all three germ layers, indicating that they are pluripotent [10]. Although P19 ECCs, EpiSCs and ESCs present similar *Oct4* expression levels, P19 ECCs and EpiSCs express lower levels of *Nanog* than ESCs [11,12]. Furthermore, P19 ECCs also share other similarities with EpiSCs, such as the preferentially use of the *Oct4* proximal enhancer [13]. In contrast to P19 ECCs, F9 ECCs and ESCs preferentially use the *Oct4* distal enhancer [13]. In addition, F9 ECCs showed levels of *Nanog* expression similar to those observed in ESCs. These observations suggest that P19 and F9 ECCs resemble different pluripotent states, a feature that has been successfully used in cell fusion reprogramming experiments to decipher the mechanisms underlying cellular pluripotency and reprogramming [14,15].

In the current study, we investigated the relationship between the pluripotency state and the reprogramming potential. To this end, we used a cell fusion protocol in which distinct pluripotent cell types were used as fusion partners. We found that EpiSCs and P19 ECCs typically exhibit a lower reprogramming potential than ESCs and F9 ECCs respectively, demonstrating that cell types presenting naïve pluripotency have a higher reprogramming potential. We also observed that the overexpression of *Sox2*, which is expressed in lower levels in EpiSCs and P19 ECCs compared with ESCs and F9 ECCs, leads to a dramatically increased reprogramming capability in EpiSCs and P19 ECCs. These findings suggest a close relationship between the pluripotency state and the reprogramming capacity, with *Sox2* levels playing a determinant role on the reprogramming potential.

## Results

### EpiSCs exhibit a low reprogramming potential

**Figure 1:**
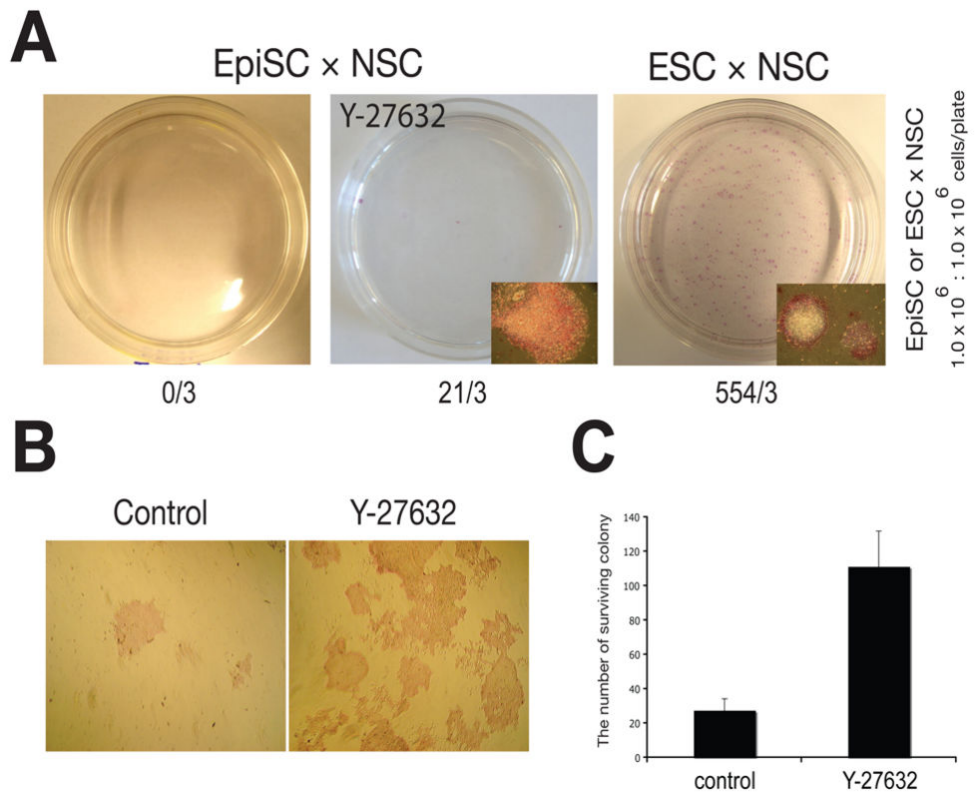
EpiSCs exhibit a low reprogramming potential. (**A**) Reprogramming potential of EpiSCs and ESCs was determined by comparing the colony-forming rate of the fusion hybrids between NSCs and EpiSCs or ESCs, respectively, in the absence or presence of the ROCK inhibitor (Y-27632). Data is represented as the number of total colonies from 3 different experiments. (**B** and **C**) EpiSCs completely dissociated by trypsinization were cultured in the presence or absence of the ROCK inhibitor, and the survival rate was assessed by counting AP-positive fusion hybrid colonies. Data is represented as mean +/- SEM.

ESCs and EpiSCs exhibit features of pluripotency, as evidenced by the ability to differentiate into cell types of all three germ layers [1,3]. Though previous reports have thoroughly characterized the potential of ESCs to reprogram somatic cells using cell fusion [14,15], the reprogramming potential of EpiSCs have not been assessed yet. Therefore, we first compared the reprogramming potential of EpiSCs and ESCs after each had been fused with neomycin-resistant NSCs. Following neomycin selection of the fusion hybrids for one week, the rate of colony formation was determined using AP staining. While fusion of NSCs with ESCs led to the production of many viable fusion hybrid colonies, no viable colonies had formed after the fusion of NSCs with EpiSCs (Figure 1A). Thus, we next tried to elucidate the mechanism underlying the extremely low reprogramming potential of EpiSCs. As EpiSCs and human ESCs grow under very similar culture conditions and need to be passaged as small cell clumps, not as single cells, we postulated that the reprogramming efficiency of EpiSCs may be difficult to quantify by using the cell fusion protocol, which requires complete dissociation of EpiSCs into single cells [1,3,14]. To increase the survival rate of the completely dissociated EpiSCs, we repeated the fusion experiment in the presence of ROCK inhibitor (Y-27632), which inhibits apoptosis and thus allows human ESCs to be grown as single cells [16]. A 3.6-fold increase in the number of EpiSCs colonies was observed in the presence of the ROCK inhibitor after complete dissociation into single cells (Figure 1B and 1C). We next fused EpiSCs with NSCs in the presence of the ROCK inhibitor and subsequently observed a few AP-positive fusion hybrid colonies (Figure 1A). These results indicate that EpiSCs indeed have a reprogramming potential ability, although it is extremely low in comparison to that of ESCs. These results suggest that although EpiSCs are pluripotent stem cells, their reprogramming potential differs from that of ESCs.

### Low reprogramming potential is a general feature of epiblast-derived pluripotent stem cells

**Figure 2:**
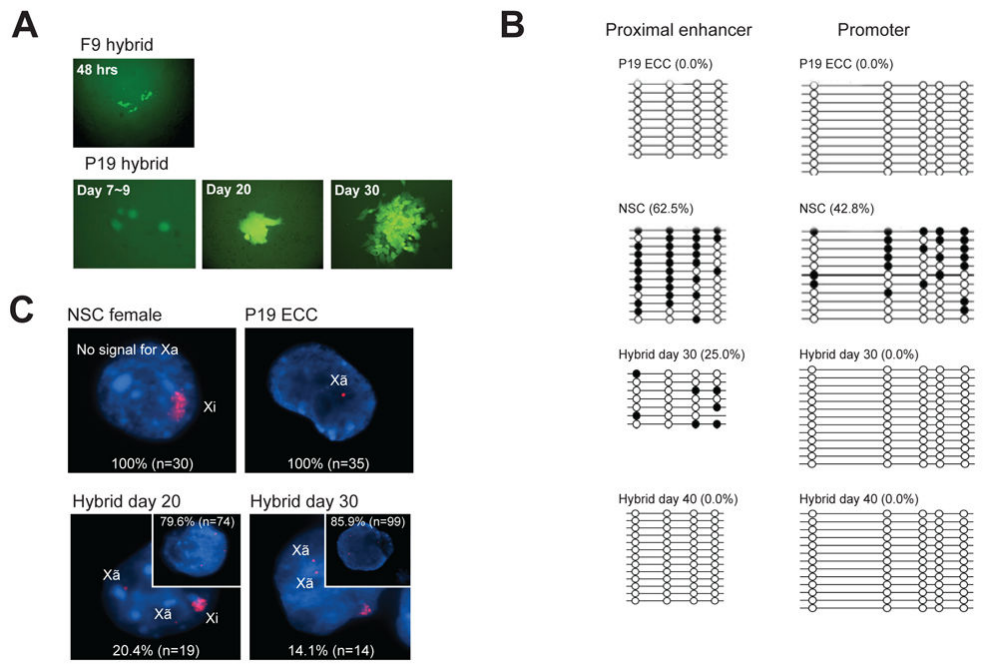
Low reprogramming potential of mouse epiblast-derived pluripotent stem cells. (**A**) Reprogramming potential of F9 and P19 ECCs was determined by monitoring the time required to reactivate the *Oct4*-GFP transgene in the reprogrammed somatic genome. (**B**) DNA methylation state of the *Oct4* regulatory regions (promoter and proximal enhancer) in P19 hybrids was analyzed using the bisulfite sequencing method. Open and filled circles indicate unmethylated and methylated CpGs, respectively. (**C**) X chromosome state was determined by *Xist*/*Tsix* RNA FISH. DNA was counterstained with 4,6-diamidino-2-phenylindole (DAPI; blue). *Xist*/*Tsix* RNA was detected as a large signal in inactive X chromosome (Xi), a pinpoint signal in active X chromosome (Xã; pluripotent cell type), and no signal in active X chromosome (Xa; somatic cell type). In P19 hybrids, the large signal (typical Xi) of female NSCs gradually changed into pinpoint signals, indicating that the X chromosome of the somatic cell genome had been reactivated as in pluripotent P19 ECCs.

Next, we tried to exclude that the extremely low reprogramming potential of EpiSCs was due to low survival after dissociation, even in the presence of the ROCK inhibitor. To this end, we investigated the reprogramming potential of P19 ECCs since P19 ECCs and EpiSCs exhibit similar features [10,13,17]. P19 ECCs can be dissociated into single cells without affecting survival, thus cell fusion experiments using P19 ECCs could be used as a model to test whether the low reprogramming potential of EpiSCs is a general feature to all epiblast-derived pluripotent cells or specific to EpiSCs. As control, we used F9 ECCs that resemble ESCs [17]. We compared the reprogramming potential of F9 ECCs with that of P19 ECCs after cell fusion with NSCs containing a green fluorescent protein (GFP) reporter gene driven by the *Oct4* promoter. The reprogramming potential was evaluated by monitoring the time required to activate the *Oct4*-GFP reporter gene expression from the reprogrammed somatic genome. Consistent with our previous findings [14], *Oct4*-GFP–positive cells were first detected in F9 fusion hybrids within 2 days post-fusion. However, *Oct4*-GFP expression became activated in P19 ECCs within at least 7 to about 9 days after fusion (Figure 2A). Moreover, while fusion of NSCs with F9 ECCs resulted in the demethylation of the *Oct4* regulatory regions within 2 days post-fusion [14], the *Oct4* proximal enhancer retained its methylated state in P19 fusion hybrids even 1 month post-fusion (Figure 2B). X chromosome reactivation is a well-known epigenetic event occurring after somatic cell genome reprogramming that can be monitored using fluorescence in situ hybridization (FISH) against the *Xist*/*Tsix* RNA. In somatic cells, the inactive X chromosome presents a large FISH signal (Xi) while the active X chromosome does not present any signal (Xa). However, both X chromosomes from female ESCs and the single X chromosome from the male ESCs are active but present a pinpoint FISH signal (Xã), suggesting a basal *Xist*/*Tsix* transcription that is specific for the naïve pluripotent state [18]. Interestingly, X chromosome reactivation was completed in F9 hybrids within 1 week of fusion [19], but not completed in P19 hybrids even at 1 month post-fusion since a large Xi signal can be still detected in 14.1% of the hybrids (Figure 2C). Collectively, these findings demonstrate that a very low reprogramming potential of both EpiSCs and P19 ECCs appears to be a general feature of mouse epiblast-derived pluripotent cells.

### Low levels of Sox2 are responsible for the low reprogramming potential of epiblast-derived pluripotent stem cells

**Figure 3:**
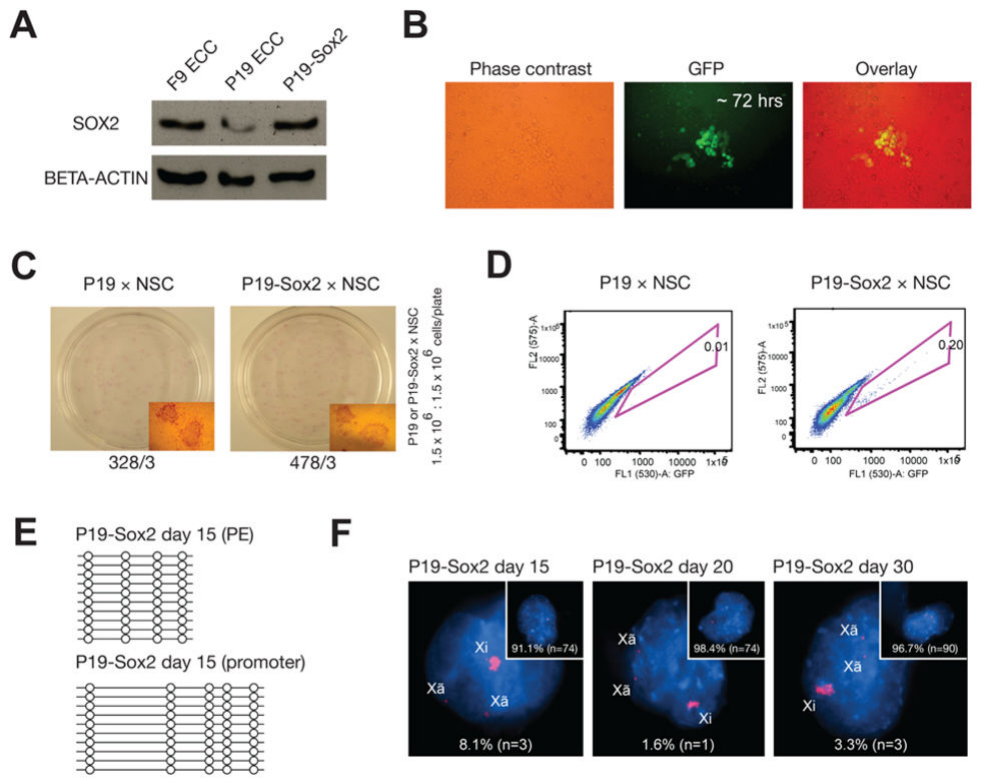
Low levels of *Sox2* are responsible for the low reprogramming potential of P19 ECCs. (**A**) After *Sox2* overexpression, SOX2 protein levels were assessed by Western blot. BETA-ACTIN was used as a loading control. (**B**) The timing of *Oct4*-GFP activation was monitored under fluorescence microscopy in P19 fusion hybrid cells overexpressing *Sox2*. (**C** and **D**) The colony-forming rates (**C**) and reprogramming rates (**D**) were determined by AP staining and FACS, respectively. Data is represented as the number of total colonies from 3 different experiments. (**E**) The reprogramming patterns of the *Oct4* regulatory regions (proximal enhancer and promoter) were analyzed in P19-*Sox2* hybrids on day 15 post-fusion by bisulfite sequencing. Open and filled circles indicate unmethylated and methylated CpGs, respectively. (**F**) X chromosome state in P19-*Sox2* hybrids was determined by *Xist*/*Tsix* RNA FISH on days 15, 20, and 30 post-fusion. DNA was counterstained with DAPI (blue). Each panel shows the percentage of non-reprogrammed cells, depicting an X chromosome state consistent with the somatic cell type (a large signal), while the small window shows the percentage of reprogrammed cells, whose X chromosome state is consistent with the pluripotent cell type (three pinpoint signals).

Although OCT4 protein levels are similar in ESCs and EpiSCs, SOX2 and NANOG levels are lower in EpiSCs than in ESCs as described previously [11]. In agreement with this observation, *Oct4* expression was similar in the cell lines tested (ESCs, and F9 and P19 ECCs) but *Nanog* and *Sox2* expression was slightly lower in P19 ECCs than in F9 ECCs, as determined by real-time RT-PCR (Supplementary information, Figure S1A). Low SOX2 levels were confirmed by Western blot (Figure 3A). To assess the effect of *Sox2* expression on genomic reprogramming, we first overexpressed *Sox2* in P19 ECCs, and confirmed the increase in *Sox2* expression by measuring mRNA and protein levels (Supplementary information, Figure S1B and Figure 3A), and then fused these *Sox2*-overexpressing P19 ECCs, which formed colonies with a slightly compact shape (Supplementary information, Figure S1C), with NSCs. Following neomycin selection, *Sox2*-overexpressing P19 ECCs (P19-Sox2) led to activated *Oct4*-GFP transgene expression within 72 hrs post-fusion (Figure 3B). Considering that wild-type P19 ECCs activate *Oct4*-GFP transgene expression after only at least 1 week post-fusion (Figure 2A), it appears likely that delayed reprogramming of P19 hybrids could be rescued by *Sox2* overexpression. After neomycin selection for 1 week, *Sox2*-overexpressing P19 hybrids (P19-*Sox2* hybrids) showed a 1.5-fold increase in the number of AP-positive hybrid colonies, indicating that *Sox2* overexpression led to an increase in the colony formation rate (Figure 3C). Moreover, while the proportion of *Oct4*-GFP–positive reprogrammed cells in the control P19 hybrids was 0.01%, the reprogramming rate in the P19-*Sox2* hybrids was 0.2%, representing an approximate 20-fold increase due to *Sox2* overexpression (Figure 3D). These P19-*Sox2* hybrids exhibited demethylation of the *Oct4* regulatory regions (Figure 3E) earlier than P19 hybrids as well as almost complete X-chromosome reactivation by day 20 post-fusion (98.4%) (Figure 3F), versus the less complete X-chromosome reactivation (85.9%) and delayed demethylation on the *Oct4* proximal enhancer element in control P19 hybrids at 1 month post-fusion (Figure 2B and 2C). In summary, these findings suggest that *Sox2* is a key factor in cell fusion–mediated reprogramming and that *Sox2* overexpression restores the reprogramming potential of P19 ECCs to a level similar to that of F9 ECCs.

### The role of Sox2 in reprogramming is not restricted to epiblast-derived pluripotent stem cells

**Figure 4:**
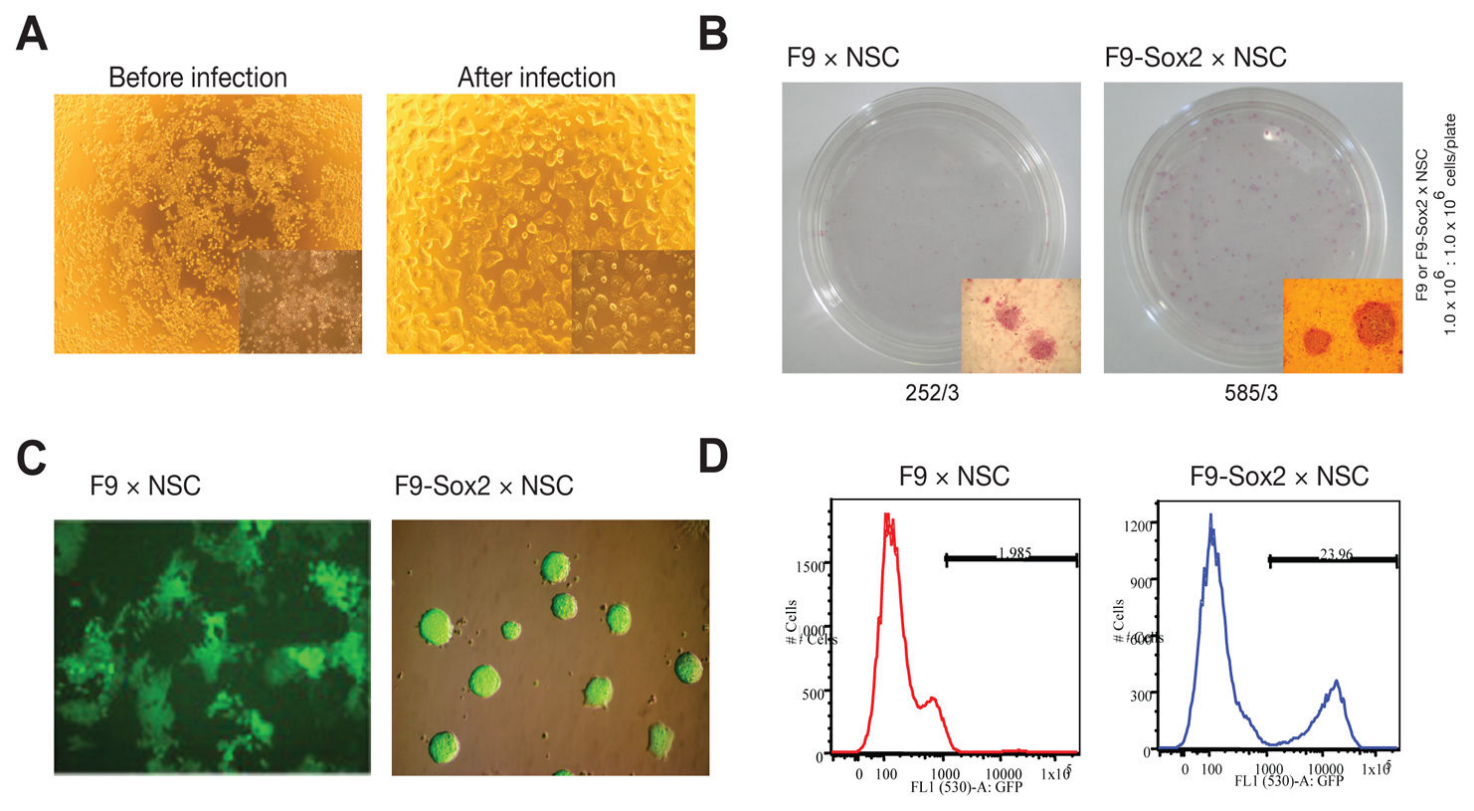
Role of *Sox2* on the reprogramming potential of cells from a non-epiblast origin. (**A**) F9 ECCs overexpressing *Sox2* exhibited a different morphology and presented more compact and round-shaped colonies. (**B**) The colony-forming rate was determined by AP staining after 1week of neomycin selection. Data is represented as the number of total colonies from 3 different experiments. (**C**) Morphology of both F9 hybrid cells and F9-*Sox2* hybrid cells. (**D**) The reprogramming rates of both F9 and F9-*Sox2* hybrids were calculated by counting the number of *Oct4*-GFP–positive cells via FACS.

To investigate whether the role of *Sox2* in cell fusion–mediated reprogramming is also conserved in non-epiblast-derived pluripotent stem cells, we overexpressed *Sox2* into F9 ECCs that express higher levels of *Sox2* than P19 ECCs (Figure 3A). Similarly, *Sox2*-overexpressing F9 (F9-*Sox2*) hybrids (Figure 4A) exhibited an increased colony-forming rate (Figure 4B) and a 12-fold increased reprogramming rate compared with the control F9 hybrids (Figure 4D). Interestingly, the overexpression of *Sox2* in F9 ECCs and F9 hybrid cells resulted in loss of the typical colony morphology and acquisition of a compact ESC-like colony morphology (Figure 4A and 4C), as observed with some clones of *Sox2*-overexpressing P19 cells (Supplementary information, Figure S1C). Therefore, the positive effect of *Sox2* on somatic cell genome reprogramming occurs irrespective of the pluripotent stem cell type used in the fusion process.

### Sox2 overexpression boosts the low reprogramming potential of EpiSCs

**Figure 5:**
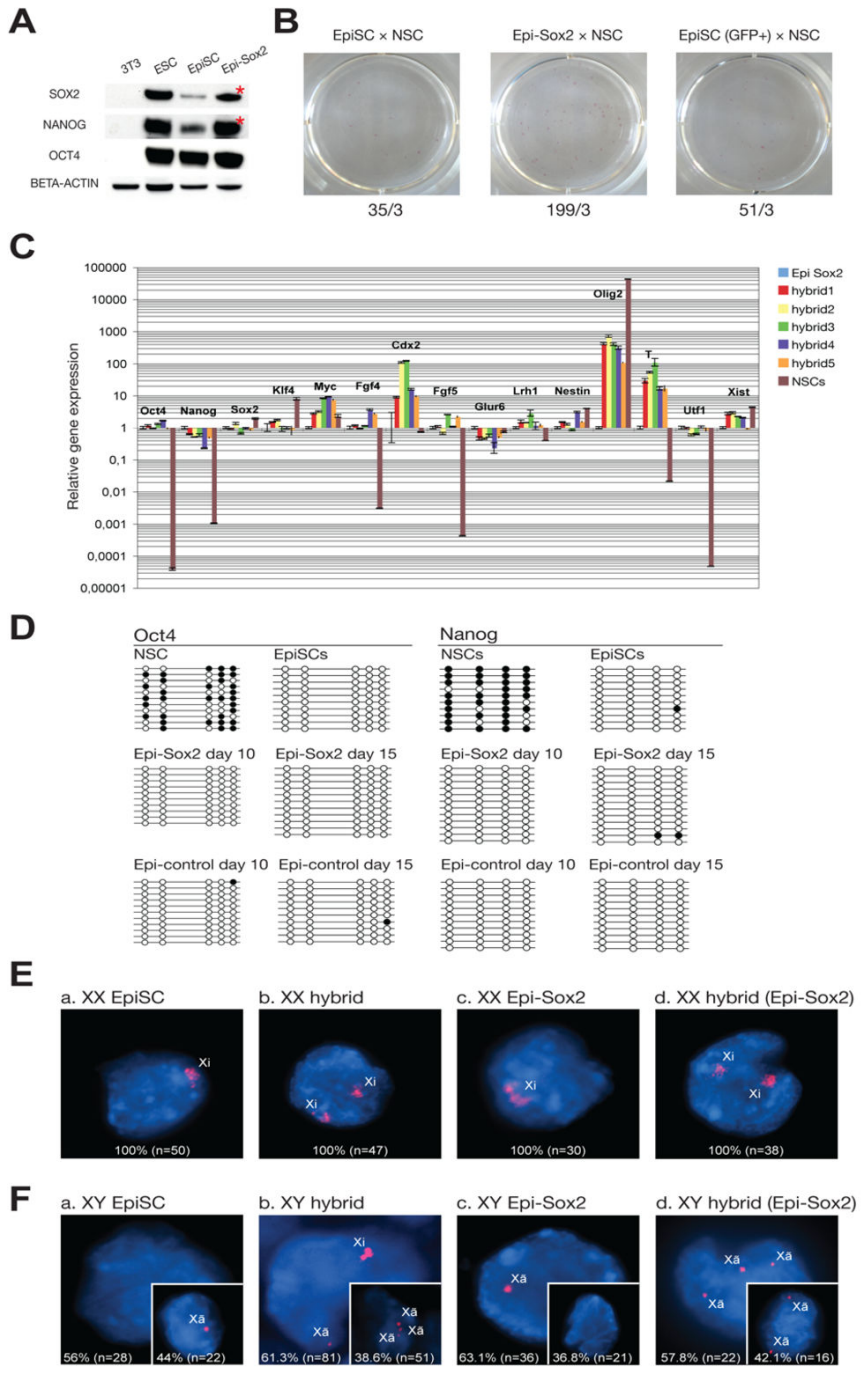
*Sox2* overexpression rescues the low reprogramming potential of EpiSCs. (**A**) OCT4, NANOG, and SOX2 protein levels were determined in Sox2-overexpressing EpiSCs (Epi-Sox2) by Western blot. (**B**) Epi-*Sox2* showed increased colony-forming rates. Data is represented as the number of total colonies from 3 different experiments. (**C**) The expression profiles of pluripotency and NSC markers in Epi-*Sox2* hybrids were assessed by real-time RT-PCR. All data are normalized to *Bact* expression and calibrated to EpiSCs, whose expression is considered 1 for all genes. The *y*-axis value is on logarithmic scale, and minor gridlines are 1/10th the value of each major gridline. Data is represented as mean +/- SEM. (**D**) DNA methylation state of *Oct4* and *Nanog* promoter regions in control hybrids and Epi-*Sox2* hybrids was compared by bisulfite sequencing. Open and filled circles indicate unmethylated and methylated CpGs, respectively. (**E** and **F**) X chromosome state was determined by *Xist*/*Tsix* RNA FISH in XX (**E**) and XY (**F**) EpiSC hybrids and Epi-*Sox2* hybrids on day 7 post-fusion. DNA was counterstained with DAPI (blue). *Xist*/*Tsix* RNA was detected as a large signal in Xi (inactive X chromosome), a pinpoint signal in Xã (active X chromosome; pluripotent cell type), and no signal in Xa (active X chromosome; somatic cell type).

Although the epiblast-derived stem cell P19 ECCs have a lower reprogramming potential than F9 ECCs and ESCs, P19 ECCs can still reprogram somatic cells with relatively higher efficiency than EpiSCs (Figure 1A). The mRNA levels of *Sox2* and *Nanog* are similar in ESCs and EpiSCs, as shown by microarray [3]. Surprisingly, SOX2 and NANOG protein levels are lower in EpiSCs, as determined by Western blot (Figure 5A). To assess whether the extremely low reprogramming potential of EpiSCs is due to low SOX2 protein levels, we overexpressed *Sox2* in EpiSCs (Epi-*Sox2*), as confirmed by increased SOX2 protein levels in EpiSCs (Figure 5A). NANOG levels were also increased, and nearly comparable to ESC levels (Figure 5A). We next investigated whether *Sox2* overexpression could also rescue the extremely low reprogramming potential of EpiSCs, as observed for F9 and P19 hybrid cells. To this end, we fused the Epi-*Sox2* cells with NSCs (Figure 5A and 5B) in the presence of the ROCK inhibitor. While control EpiSCs produced a few hybrid colonies, Epi-*Sox2* cells produced about 5 to 6 times more hybrid colonies (Figure 5B). We next evaluated whether the EpiSC fusion hybrid cells had undergone successful reprogramming. First, we analyzed the gene expression profile of 5 hybrid lines, which were randomly picked from Epi-*Sox2* hybrid cell colonies (Figure 5C). As expected, Epi-*Sox2* hybrids exhibited a gene expression pattern consistent with successful reprogramming into an EpiSC state. After fusion, there was upregulation of the pluripotency marker genes *Oct4* and *Nanog*, with levels comparable to those of EpiSCs, and of the EpiSC markers *Fgf5* and *T*. The control hybrids also showed gene expression patterns similar to those of Epi-*Sox2* hybrid cells (data not shown). We next analyzed the epigenetic reprogramming of both control and Epi-*Sox2* hybrid cells by monitoring the DNA methylation state of the *Oct4* and *Nanog* regulatory regions in a time-course manner. While the *Nanog* and the endogenous *Oct4* promoter regions were highly methylated in NSCs, both regions were completely demethylated in control and Epi-*Sox2* hybrids within 10 days post-fusion (Figure 5D), indicating that the reprogramming potential of EpiSCs is very low, but once EpiSCs are fused with somatic cells, they can induce normal reprogramming in somatic cells. X-chromosome reactivation was also analyzed in both XX and XY EpiSC hybrids. EpiSCs are supposed to show the somatic cell forms of the X chromosome (one large signal in XX / no signal in XY). As expected, XX EpiSCs have an inactive X chromosome (Figure 5E-a). Surprisingly, almost half of the XY EpiSCs (44%) have the pluripotent cell form of the X chromosome (Xã), as they showed only one pinpoint signal, a common characteristic of XY ESCs (Figure 5F-a). *Sox2*-overexpressing EpiSCs (XX and XY) showed the same X chromosome state as control EpiSCs (Figures 5E-c and 5F-c). XX hybrids from both EpiSCs and Epi-*Sox2* hybrid cells exhibited a non-reprogrammed X chromosome pattern, as they showed two inactive (large signals) and two active (no signals) X chromosomes, indicating that XX EpiSCs have no X-chromosome reactivating potential (Figures 5E-b and 5E-d). On the other hand, approximately 38% of XY EpiSC hybrids showed evidence of X-chromosome reactivation, as the X chromosomes of XX NSCs (a large and no signal) were reprogrammed into two or three pinpoint signals within 7 days post-fusion (Figure 5F-b). However, the rest of the XY EpiSC hybrids (61%) still exhibited the non-reprogrammed NSC X-chromosome pattern (Xi). Thus, only a subpopulation of XY EpiSCs is X-chromosome reactivating competent (Figure 5F-b). Unexpectedly, we could not detect the inactivated NSC X-chromosome (Xi) in XY Epi-*Sox2* hybrids within 7 days post-fusion, indicating that *Sox2* overexpression enhances the X reactivation potential of the XY EpiSCs (Figure 5F-d). Previously, we have reported that EpiSC cultures are heterogeneous and comprise two distinct subpopulations, *Oct4*-GFP–positive and –negative EpiSCs, which are in a state of dynamic equilibrium [11]. *Oct4*-GFP–positive and –negative EpiSCs correspond to *in-vivo* epiblasts of an early and late developmental stage, respectively, in terms of gene expression patterns, epigenetic feature, specific *Oct4* enhancer activity, and functional pluripotency. *Oct4*-GFP–positive EpiSCs present a higher degree of pluripotency, as they can contribute to chimera formation after blastocyst injection. Therefore, we hypothesized that *Oct4*-GFP–positive EpiSCs may have a higher reprogramming potential than *Oct4*-GFP–negative EpiSCs. Following cell fusion, *Oct4*-GFP–positive cells produced an increased number of fusion hybrid colonies compared with control EpiSCs (Figure 5B). Again, these data show that cellular pluripotency is closely associated with reprogramming capability.

## Discussion

In the current study, we attempted to uncover any association between the pluripotent state, naïve or primed, and the reprogramming capacity as well as the effect of *Sox2* on the reprogramming potential. Although previous studies had demonstrated that the newly established EpiSCs exhibited cellular pluripotency [1,3], they had not determined the reprogramming potential of EpiSCs. To delve deeper into these issues, we fused NSCs with either ESCs or EpiSCs, and found that ESCs produced many more viable fusion hybrid colonies than EpiSCs. Likewise, P19 ECCs, which have an epiblast origin [13], also showed a delayed and a lower reprogramming potential than the non-epiblast-derived F9 ECCs. Thus, our results demonstrate that a low reprogramming potential is a general feature of mouse epiblast-derived pluripotent stem cells.

Both P19 ECCs and EpiSCs showed lower levels of SOX2 protein, but not OCT4, compared with F9 ECCs and ESCs, respectively. These findings indicate that although low *Oct4* levels have a detrimental effect on the viability of pluripotent cells, low *Sox2* levels have a moderate effect on the maintenance of pluripotency. Interestingly, *Sox2* overexpression in P19 hybrid cells led to a significantly increased colony-forming rate and reprogramming efficiency, as well as to a shortened time frame for epigenetic modifications—activation of pluripotency marker genes, DNA demethylation of *Oct4* regulatory regions, and X-chromosome reactivation. Although forced expression of *Sox2* was described to trigger the differentiation of pluripotent cells [20], we observed that *Sox2* overexpression induces compact colony morphology in F9 and P19 ECCs, as also previously reported [21]. EpiSCs overexpressing *Sox2* produced significantly more fusion hybrids colonies compared with control EpiSCs. These results indicate that EpiSCs, along with P19 and F9 ECCs, could acquire a higher reprogramming capacity after *Sox2* overexpression.

Undifferentiated male EpiSCs should theoretically possess X-chromosome patterns consistent with those of somatic cells. However, approximately half of the XY EpiSCs exhibit a pinpoint *Xist*/*Tsix* FISH signal, which is actually a feature of XY ESCs. EpiSCs are composed of two subpopulations of cells that represent the *in-vivo* early and late stage epiblast, as we have shown in our previous study [11]. Therefore, it is likely that EpiSCs with a pluripotent type of X chromosome correspond to an *in-vivo* epiblast of an early stage, while those with a somatic type represent an *in-vivo* epiblast of a relatively late stage. It is also likely that the conversion of the pluripotent type of X chromosome into the somatic type occurs faster in XX EpiSCs, which normally takes 24 hrs, as both XX and XY EpiSCs originate from 5.5 d.p.c. embryos, which had X-chromosome inactivation nearly completed. *Sox2* overexpression in EpiSCs also led to faster X-chromosome reactivation. While Epi-*Sox2* hybrids showed nearly complete X-chromosome reactivation within 7 days post-fusion, more than 60% of control EpiSC hybrids showed remaining inactive X chromosomes on day 7 post-fusion. In addition, the X chromosome state of control EpiSC hybrids was consistent with a non-reprogrammed state as late as day 18 post-fusion (data not shown). *Sox2* is known to bind on *Xist* and repress *Xist* expression together with *Oct4* and *Nanog* [22]. Therefore, it is highly likely that EpiSCs acquire a higher X-chromosome reactivating potential after *Sox2* overexpression.

P19 ECCs and EpiSCs express SOX2 and NANOG at lower levels than ESCs. A recent study [12] showed reversion of EpiSCs to an ESC-like state after *Nanog* overexpression. As *Sox2* overexpression led to increased NANOG protein levels, we expected a *Sox2*-mediated reversion of EpiSCs into a naïve pluripotent state [12] but this did not occur (data not shown). Considering that *Nanog* is very sensitive to dosage levels [12,15], it is very likely that the *Nanog* level indirectly induced via *Sox2* overexpression is not sufficient to revert EpiSCs to an ESC-like state. Collectively, our findings suggest that *Sox2* is a determinant of reprogramming capacity.

Cellular pluripotency can be determined by several criteria such as expression of pluripotency markers, *in vitro* differentiation potential, formation of teratomas and chimeric contribution into somatic cells and the germline. As EpiSCs with a primed pluripotency can form teratomas but not chimeras, the ability for chimeric contribution is a critical criterion for distinguishing a naïve and primed state of cellular pluripotency. However, the generation of mouse chimeras is not simple, as it requires highly experienced skills with a very complicated experimental setting. Based on our findings, ESCs and EpiSCs differ not only in chimeric contribution capacity, but also in reprogramming potential via fusion-mediated reprogramming. Therefore, our data suggest that the reprogramming potential of pluripotent stem cells could be a simple but effective indicator for distinguishing the functional state of pluripotent stem cells.

## Methods

### Cell culture and fusion

P19, F9 and 293T (ATCC) were cultured on gelatin-coated (0.1% in PBS) dishes in standard culture media: high-glucose DMEM (Gibco BRL) containing 15% fetal calf serum (FCS; Gibco BRL), 1X penicillin/streptomycin/glutamine (Gibco BRL), and 1X nonessential amino acids (Gibco BRL). ESCs were grown in standard culture media plus LIF (Chemicon). The neural stem cell (NSC) line used in this study was derived from the brain tissue of 16.5-dpc OG2/ROSA26 female mice and has been previously reported [23]. NSCs were grown in DMEM-F12 medium (Gibco BRL) supplemented with 20 ng/ml epidermal growth factor (EGF; Peprotech), 20 ng/ml bFGF (New England Biolabs), B27 supplement (Gibco BRL), 8 mM HEPES (Gibco BRL) and 1X penicillin/streptomycin/glutamine. Feeder-free EpiSCs [23] were cultured in conditioned medium (CM). For conditioning, irradiated CF-1 mouse embryonic fibroblasts (MEFs) were seeded at density of 5x10^4^ cells/cm^2^ and incubated during 24 hrs in Knockout DMEM medium (Invitrogen) containing 20% Serum Replacement (Invitrogen), 1X penicillin/streptomycin/glutamine (Gibco BRL), 1X nonessential amino acids (Gibco BRL), 1X β-mercaptoethanol (Gibco BRL) and 5 ng/ml bFGF (New England Biolabs). The CM medium was filtered, and bFGF (5 ng/ml) was added to it. For passaging feeder-free EpiSCs, colonies were incubated with Collagenase IV (Invitrogen) for 5 min at 37^°^C and triturated by using a cell scraper. Cell clumps were replated on FCS-coated dishes, with medium changes every 24 hrs.

Pluripotent cells and NSCs were fused according to our previous protocol [14]. Briefly, pluripotent cells were mixed with NSCs in a 1:1 ratio, and then washed in PBS. The mixture was centrifuged at 130g for 5 min, and 1 ml of a prewarmed 50% polyethylene glycol 1500 solution (PEG1500; Roche) was added to the cell pellet over 1 min. An additional 20 ml of DMEM was added to the cell suspension over 5 min, with constant stirring. The cells were centrifuged at 130g for 5 min to remove the PEG, washed gently with DMEM, and cultured in ECC, ESC, and EpiSC medium, respectively. The fusion hybrid cells were selected by treating neomycin (300 µg/ml) after 24 hrs post-fusion, and colony-forming rate was calculated by counting the number of colonies that stained positive for alkaline phosphate (AP, Alkaline Phosphatase Detection Kit, Chemicon) 1 week post-fusion.

### Western blot and immunocytochemistry

Western blots were performed using anti-Nanog (Cosmo Bio, REC-RCAB0002PF, 1:500), anti-Oct4 (Abcam, ab19857, 1:400), and anti-Sox2 (Abcam, ab15830, 1:3000) primary antibodies. Anti-Sox2 (Chemicon, 2003600, 1:1000) and anti-Oct4 (Abcam, ab19857, 1:500) antibodies were used for immunocytochemistry.

### *Xist/Tsix* FISH (fluorescence in situ hybridization)

Fusion hybrid cells were placed onto Roboz slides, which were then fixed in 4% paraformaldehyde in PBS for 10 min at room temperature, rinsed in 70% ethanol, and stored in 70% ethanol at 4^°^C until use in FISH. *Xist*/*Tsix* RNA was detected with a Xist RNA FISH probe that was labelled with Cy3 and that spans the entire *Xist* cDNA. *Xist*/*Tsix* RNA FISH was performed as described previously [24]. Images were obtained using applied spectral imaging (ASI) camera and analyzed with FishView software (Applied Spectral Imaging; ASI GmBH).

### DNA methylation analysis

To assess the DNA methylation status of *Oct4* and *Nanog* regulatory elements, genomic DNA was treated with sodium bisulfite to convert all unmethylated cytosine residues into uracil ones using EpiTect Bisulfite Kit (Qiagen) according to manufacturer’s protocol. Following bisulfite treatment, all the selected genomic regions were amplified using a nested primer approach. PCR amplifications were performed using SuperTaq polymerase (Ambion) in a total volume of 25 µl. All PCR amplifications consisted of a total of 40 cycles of denaturation at 94^°^C for 30s, annealing at the appropriate temperature for each target region for 30s, extension at 72^°^C for 30s with a 1^st^ denaturation at 94^°^C for 5 min, and a final extension at 72^°^C for 10 min. The primer sequences and annealing temperatures used were as follows: endogenous *Oct4* 1^st^ sense 5’- TTTGTTTTTTTATTTATTTAGGGGG-3’, endogenous *Oct4* 1^st^ antisense 5’- ATCCCCAATACCTCTAAACCTAATC-3’ (299 bp, 45^°^C), endogenous *Oct4* 2^nd^ sense 5’-GGGTTAGAGGTTAAGGTTAGAGGG -3’, endogenous *Oct4* 2^nd^ antisense 5’-CCCCCACCTAATAAAAATAAAAAAA -3’ (161 bp, 55^°^C), *Nanog* 1^st^ sense 5’-TTTGTAGGTGGGATTAATTGTGAA -3’, *Nanog* 1^st^ antisense 5’-AAAAAATTTTAAACAACAACCAAAAA -3’ (312 bp, 45^°^C), *Nanog* 2^nd^ sense 5’- TTTGTAGGTGGGATTAATTGTGAA -3’, and *Nanog* 2^nd^ antisense 5’-AAAAAAACAAAACACCAACCAAAT -3’ (188 bp, 55^°^C). For each primer set, 3 µl of product from the first round of PCR was used in the second round of PCR. The amplified products were verified by electrophoresis on 1% agarose gel. The PCR products were subcloned using the PCR 2.1-TOPO vector (Invitrogen) according to the manufacturer’s protocol. Reconstructed plasmids were purified using the QIAprep Spin Miniprep kit (Qiagen) and individual clones were sequenced (GATC-biotech, Germany). Clones were only accepted if there was at least 90% cytosine conversion and all possible clonalities were excluded based on the criteria from BiQ Analyzer software (Max Planck Society, Germany). For each fusion hybrid, results were confirmed by performing at least 10 replicates per selected genomic region and at least two separate bisulfite treatments.

### Vector construction and transduction with lentivirus vectors

pLVTHM-Sox2 was constructed by replacing the GFP in pLVTHM with the Sox2 coding sequence that was directly amplified from ESCs. pLVTHM was provided by D. Trono (Geneva, Switzerland). The recombinant lentivirus was produced by transient transfection of 293T cells with 12 µg of pLVTHM, 8.5 µg of psPax2, and 3 µg of pMD2.G using Lipofectamine 2000 (Invitrogen), according to the manufacturer’s protocol. The supernatant was collected at 24 and 48 hrs of transfection and was then concentrated by ultracentrifugation at 26,000 rpm for 2 hrs at 4°C using a SW41 rotor (Beckman Coulter). After ultracentrifugation, the supernatant was decanted, and the viral pellet was resuspended in 200 µl of DMEM. The suspension was stored at -80°C until use. Packaging plasmids were also provided by D. Trono. EpiSCs, P19 ECC and F9 ECC cells were plated on 24-well plates (5 x 10^4^ cells/well), and after 24 hrs, 20 µl of the concentrated virus was added to the medium. Cells were washed after 16 hrs of incubation. After 48 hrs, the cells were once again subjected to this procedure to increase the viral transduction.

### Flow cytometry

For FACS sorting, cells were dissociated with 0.25% trypsin EDTA (Invitrogen), neutralized with DMEM containing 10% FCS, washed with PBS, and then filtered through a 40-µm nylon mesh to remove large cell clusters. The cells were resuspended in the appropriate culture medium (5 x 10^6^ cells/ml) and analyzed using a FACSAria cell sorter (BD Biosciences). GFP-positive cells were sorted by FACS using a 100-µm nozzle. 

## Supporting Information

Figure S1 Overexpressing *Sox2* in P19 ECCs. (**A**) The expression levels of *Oct4*, *Nanog*, and *Sox2* were compared in three different cell lines: ESCs, F9 ECCs, and P19 ECCs. (**B**) *Sox2* expression was analyzed after transduction of different amount of viruses into P19 ECCs. (**C**) Comparison of the morphology of P19 ECCs (non-infected) and P19-*Sox2* cell lines (clone 1~3).
(TIF)
