# Supplementary figures and images for: Sox2 Level Is a Determinant of Cellular Reprogramming Potential
Source: PLoS One. 2013 Jun 18;8(6):e67594. doi: 10.1371/journal.pone.0067594 (PMC3688988; doi:10.1371/journal.pone.0067594)

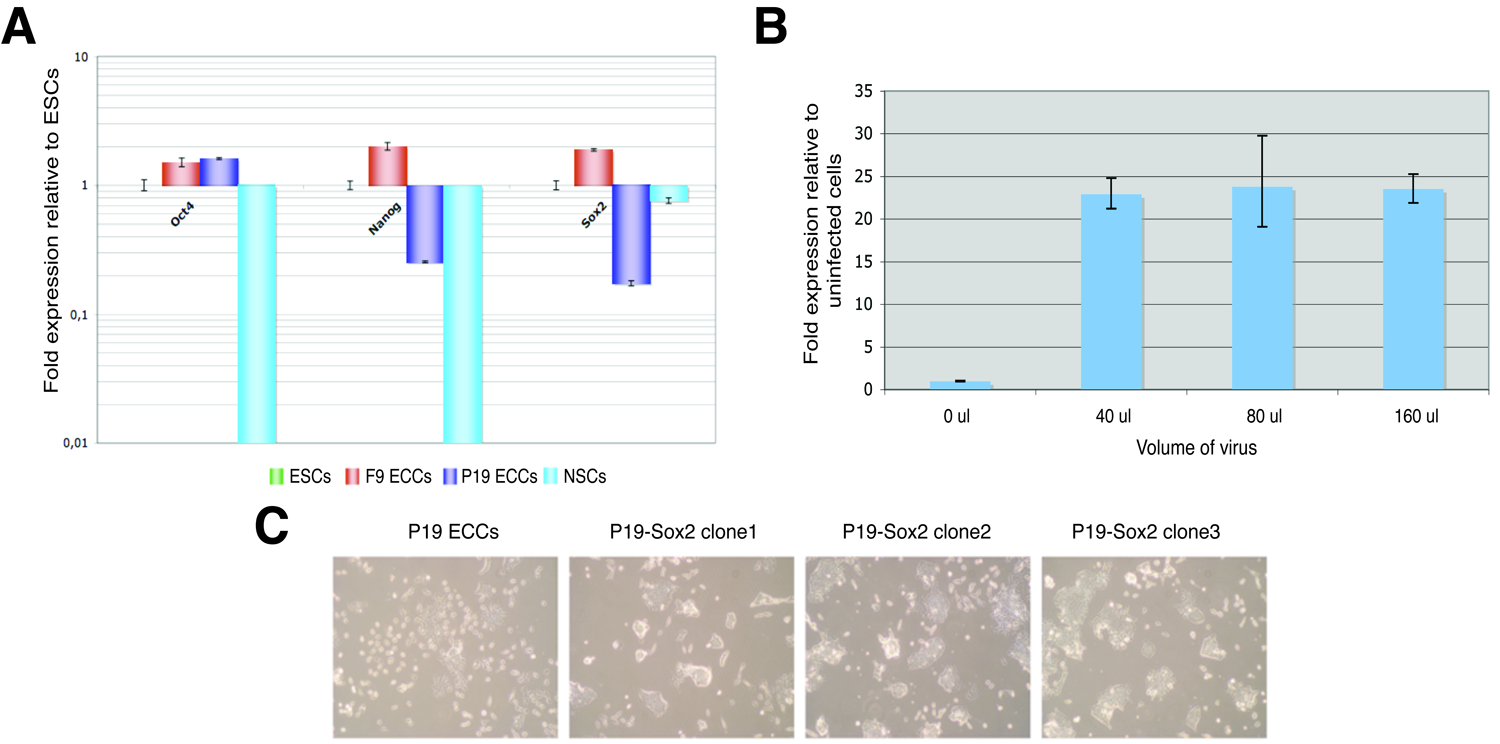

Supplement: Figure S1 — (A) The expression levels of Oct4, Nanog, and Sox2 were compared in three different cell lines: ESCs, F9 ECCs, and P19 ECCs. (B) Sox2 expression was analyzed after transduction of different amount of viruses into P19 ECCs. (C) Comparison of the morphology of P19 ECCs (non-infected) and P19-Sox2 cell lines (clone 1~3). (TIF) [file pone.0067594.s001.tif]
